# Going Green Post COVID-19: Employer Perspectives on Skills Needs

**DOI:** 10.1177/02690942231151638

**Published:** 2023-01-27

**Authors:** Sarah Strachan, Alison Greig, Aled Jones

**Affiliations:** Global Sustainability Institute, 2369 Anglia Ruskin University, Cambridge, UK

**Keywords:** green skills, green jobs, post COVID-19, low-carbon transition

## Abstract

Achieving a just transition to a low carbon economy and society, in the wake of the COVID-19 pandemic, is arguably one of the greatest policy challenges facing governments. It is also of deep concern to businesses, employees and the organisations that represent them. Much of the focus, particularly at policy level, has been on the potential of this transition to create new jobs especially through the growth of renewable energy and clean technology. In this paper, we argue that this focus on ‘green jobs', and in particular new green jobs, grossly underestimates the skills needs of a future workforce able to deliver a transition to a more sustainable low-carbon economy. The focus of this study is to gain an understanding of what skills are required to support the transition beyond these sectors. It critically reports on the results of a series of in-depth interviews with senior managers in key organisations within Cambridgeshire and Peterborough, UK. It sheds a light on the significant employment transitions taking place in organisations who are not specifically focused on delivering ‘green’ products or services. It finds widespread acknowledgement of the importance of a green recovery, albeit predicated by economic growth. The key skills needs reported, at all levels were likely to be ‘soft’ transferrable skills rather than ‘hard’ technical skills. COVID-19 was recognised as both a disrupter and as a catalyst for a green transition.

## Highlights

• Focusing on ‘green jobs' underestimates labour market changes for low carbon transition
• Green skills are being developed across all sectors and jobs
• Interpersonal skills are often as important as technical skills
• COVID-19 disrupted low carbon transition but may be a positive in longer term

## Introduction

Achieving a successful transition to a low carbon economy is one of the greatest policy challenges facing governments and their employers worldwide, and is of deep concern to businesses, workers and the organisations that represent them (ILO, 2011). The COVID-19 crisis and the resulting global slowdown in economic activity created significant additional social consequences, as well as barriers and opportunities within the context of this transition. To simultaneously address the UK’s climate commitments and 2050 net zero goal, as well as the need for economic recovery following COVID -19, the UK government has committed to a recovery which is also ‘green’. So far, its focus has been largely on the creation of new ‘green jobs' associated with carbon reducing technologies, such as carbon capture, hydrogen, nuclear, electric vehicles, and renewable energy. Massive public investments have been proposed to promote green growth and green jobs, yet little is known about their impacts on labour market outcomes; two recent papers by Chen, et al., 2020 and Popp, et al., 2020 acknowledge this lack of research.

In this study, we respond to this gap in the research and question ‘what are the skills required to support an inclusive low carbon, post-covid-19 transition to a cleaner fairer economy and society?' The focus for this study is on one specific region of the UK – Cambridgeshire and Peterborough – a large and diverse region with significant inequalities in skills and economic outcomes (CPCA, 2019). This diversity of issues in conjunction with the scope of this research to incorporate the skills required across organisations, by sector and role in the transition provides a basis for the generalisability of the findings from this study to other regions in the UK and further afield. By engaging with global, national and local organisations, beyond the usual focus of green jobs or skills required in the environmental goods and services sector (EGSS), this research provides a broad understanding of what skills are required to support the transition at different stages and across sectors.

## A review of the literature

This study was developed in the context of a targeted review of the international literature and UK policy. Aldersgate (2020), note that government efforts, and the public and business support for a green recovery requires not only policies to drive low-carbon economic activity, but also investment in people and skills. Accurate and detailed labour market information is critical for policymakers as they navigate rapid, complex, and uncertain shifts in the economy and society. Skills shortages, in particular, are notoriously difficult to measure, hidden from view as companies work around problems by increasing the workload of existing employees, outsourcing work to other organisations or even adapting their product market strategies so that they are less dependent on a specifically skilled workforce.

### Skills for the future

Skill shortages in sectors that are essential for our transition to net-zero, such as renewable energy generation, have been the focus of most research. For example, Bakhshi et al. (2017) detail how these sectors are experiencing a variety of skills shortages. Back in 2011, the International Labour Organisation (ILO, 2011) noted that the economy’s net zero transition is only going to be possible if workers can adapt flexibly and transfer from areas of decreasing employment to new and emerging industries. Almost a decade later, the World Economic Forum (WEF, 2020) note that the window of opportunity to reskill and upskill workers from so called ‘brown sectors’ had become shorter in the newly constrained labour market.

As well as the literature on the role of green skills in the low carbon transition to a cleaner, fairer economy and society, we also need to acknowledge that there are disruptors, like COVID-19 and Brexit, to prevailing economic and societal dynamics. In the UK Commission for Employment and Skills' study (Störmer, et al., 2014) the authors outline a number of these as ‘disruptive scenarios’ looking ahead to 2030. These include the following: the great divide, skills activism, innovation adaptation, the shrinking middle and the four generational workplaces (Störmer, et al., 2014).

Globally, a number of studies have explored the employment impact of the emerging ‘green’ economy (Cai, et al., 2011) and many have set up scenarios to simulate employment impacts of possible green economy scenarios in 2020, 2030 or even 2050 (see Kühn et al., 2018). However, once again most focus on renewable and new energy (Lehr et al., 2012), which is only one of many aspects of a green economy (Cai et al., 2011). The World Bank’s policy research working paper, ‘Green’ Growth, ‘Green’ Jobs and Labour Markets, concludes that *‘...a policy-induced structural change of this sort…requires attention to active labour market policies with respect to training, education, and transitional income support’* (Bowen, 2012 p. 35).

There is also a spatial dimension to these changes. The ILO notes that the green transition will be a significant challenge for regions with a major concentration of ‘brown sector’ industries, and for individuals currently working in these industries, while regions with a major concentration of ‘green sector’ industries will flourish. As Kapetaniou and McIvor (2020) point out, transition towards a low carbon and environmentally less destructive economy in the UK will lead to significant changes across many sectors and occupations. They also warn that this transition could also increase inequalities between individuals, industries and regions.

Although jobs may be lost or transformed in the brown sector, the ILO suggests that the so-called ‘greening' of the labour market will create new jobs in the green sector that could produce employment gains and prevent net job losses (ILO, 2015). As Bakhshi et al., (2017) point out, none of this will be possible without a consideration of education and training curricula, including apprenticeships and workplace training, careers guidance, occupational standards, migration and social insurance.

### Terminology

Kapetaniou and McIvor (2020) include a definition of ‘green’ and ‘brown’ sectors based on their level of carbon emissions. The transition to a ‘low carbon’ economy refers to improvements in energy efficiency, substitution of renewable energy for carbon-emitting forms of energy, and more broadly to the decarbonisation of economic activity. In terms of jobs, ‘green’ can have a broader concept, also encompassing other sustainability issues, such as water conservation and prevention and remediation of pollution (ILO, 2011). Green skills are broader still, referring to skills in the low carbon and environmental goods and services sector, and also those needed to help all businesses transition to a low carbon and sustainable future (BIS, 2011)

The terms ‘low carbon’ and ‘green’ are often used interchangeably in the literature, both are included in the scope of this study in the context of understanding the skills that will be required to help support a low carbon transition to a cleaner, fairer economy and society (see also Consoli et al., 2016 who analyse the difference in skills needs between green and non-green jobs).

**Figure 1.**
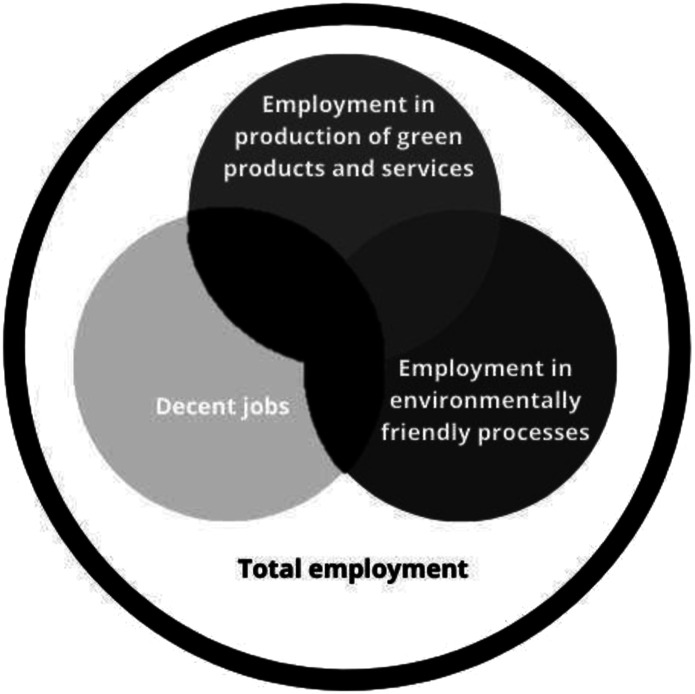
Green jobs are those in the areas shaded black (adapted from ILO, 2016).

Research literature and policy documents often also use descriptors such as ‘green jobs’ and ‘green skills’ interchangeably. The ILO (2016) recognise that green jobs must also be ‘decent’ jobs, acknowledging the key socially inclusive element of these jobs. They also make a distinction between employment in green economic sectors from an output perspective, and job functions in all sectors from an environmentally friendly process perspective (Figure 1).

Whilst this ILO’s definition of green jobs is comprehensive, much of the focus has been on new jobs created within companies providing environmental goods and services. These so-called Environmental Goods and Services Sector (EGSS) are represented by the upper circle in Figure 1 and potentially misses the significant transformation taking place beyond this. For example, to what extent do existing jobs need to be ‘environmentally friendly’ to be counted is not clear. This circle potentially includes all jobs in all sectors, even those considered high carbon brown sectors as they refocus their activity and outputs to address the challenges and opportunities of net zero. In this research, we explore the boundaries of this circle from the perspective of employers.

We contend that placing an emphasis on skills rather than jobs is more useful both in this study and perhaps the wider economy in order to understand the dynamics of the transition taking place and the challenges and opportunities this presents. In the context of this research ‘green’ skills refer to all the knowledges, skills, competencies and attributes required in the workforce to support a low carbon transition to a cleaner, fairer economy and society or more colloquially, ‘going green’ and removes the association with particular roles, functions or sectors.

The European Centre for the Development of Vocational Training (CEDEFOP, 2018) suggest that a balance of generic skills (e.g. autonomy and communication), generic green skills (such as reducing waste and improving energy and resource efficiency) and ‘topping up’ existing job-related skills is much more important to developing a low-carbon economy than more specialised, green skills.

**Figure 2.**
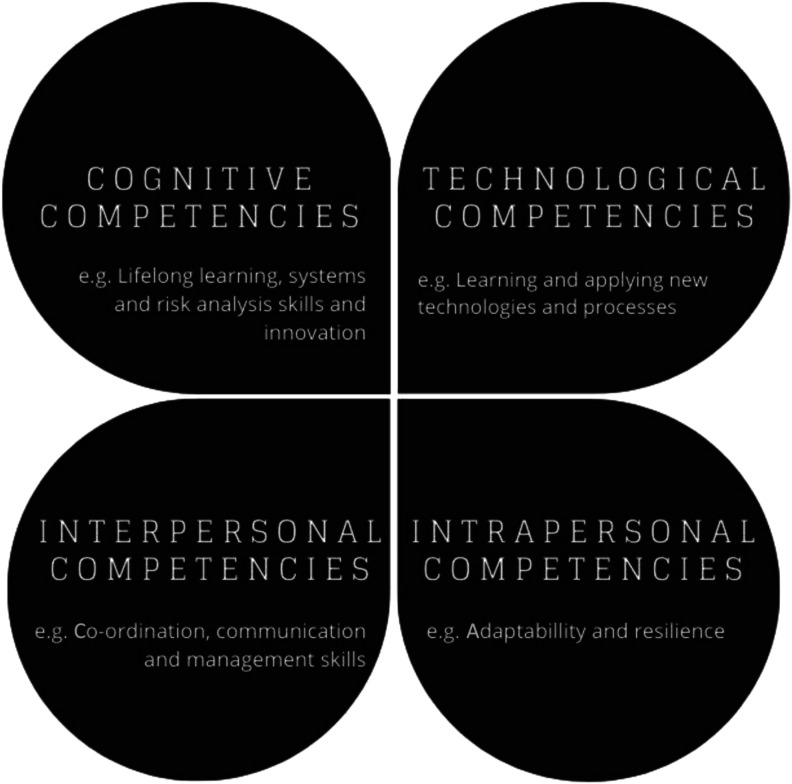
Skill needs for the low carbon economy. (Adapted from CEDEFOP, 2018). Model of Green Skills Competency (adapted from Pavlova, 2016).

In the context of the technical and vocational education and training (TVET) literature, in a study of the perspective of employers and academics, Zolkifli and colleagues conclude that TVET could serve as an effective platform in promoting generic green skills (Zolkifli, et al., 2016). In addition, some of the literature advocates a particular emphasis on the development of values and attitudes to achieve greening. Margarita Pavlova’s (2016) conceptual framework – the Model of Green Skills Competency – balances four competencies; cognitive competency, technology competency, intrapersonal competency, and interpersonal competency (Figure 2).

This competency-based approach provides a framework for this study, particularly when used in conjunction with the mapping exercise undertaken by Bakhshi et al., in the *Future of Skills* report (2017) and segmentation of UK sectors outlined in the *Going Green* report (Kapetaniou and McIvor, 2020). In the absence of a UK specific skills taxonomy, acknowledged as one of the current priorities of the new Unit for Future Skills (UFS) (UK Government, 2022), Bakhshi et al., utilises the Occupational Information Network (a program of the U.S. Department of Labor known as O*NET used to categorise occupations based on a wide array of data from workers) which includes knowledge, skills, abilities and work that needs to be performed.

In summary, whilst the literature acknowledges the importance of an appropriately skilled workforce to a low carbon transition to a cleaner, fairer economy, and society, there is a paucity of data on how green skills are defined, what upskilling, and reskilling will be required and who will be responsible for ensuring this happens. Therefore, the focus for this study is on this investment in people and specifically on addressing the research question of ‘what are the skills required to support an inclusive low carbon post-COVID-19 transition to a cleaner, fairer economy and society?'

## Material and method

This study is situated in a specific region in the UK – Cambridgeshire and Peterborough. It is an area defined by three distinct economies – Greater Cambridge, Greater Peterborough and the Fens – with different sector specialisms and therefore differing social and economic skills needs (CPCA, 2019). Broadly speaking Greater Cambridge has the highest levels of skills and best educational outcomes; Greater Peterborough and the surrounding area experiences lower levels of employment and greater economic inactivity and the Fens has lower labour market performance, related to the accessibility of both jobs and training. Although the area is home to large and globally significant businesses, small/medium businesses dominate as is the case in many areas of the UK and beyond. Therefore, this study may also offer insights into more generalisable issues such as dependence on growth, equity and fairness of decarbonisation and resilience of industry sectors and communities which are challenging policymakers and business leaders throughout the UK and worldwide.

**Figure 3.**
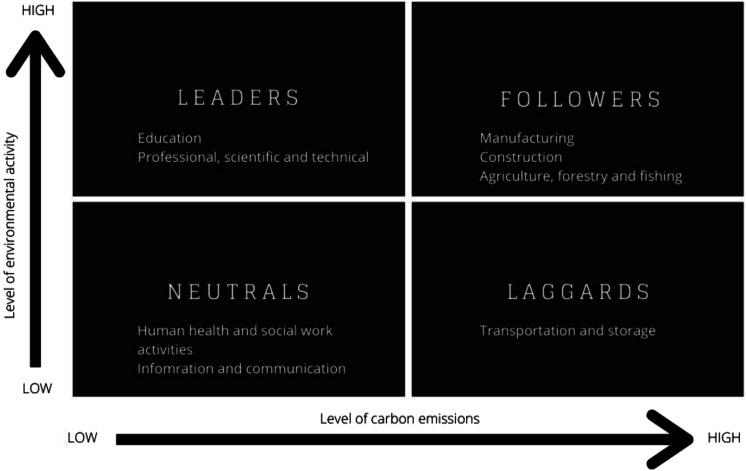
Eco-Transformation of Industries Matrix with sectors highlighted by participant (adapted from Kapetaniou and McIvor, 2020).

Taking a predominantly qualitative approach to data collection and analysis, this study used interviews with organisational leaders. The methodology is developed in the context of approaches taken in *The Future of Skills* report (Bakhshi et al., 2017) as outlined above in the literature review and participants selected based on their sector’s role in the transition, as outlined in the *Going Green* report (Kapetaniou and McIvor 2020) (See Figure 3 below). Utilising Kapetaniou and McIvor’s Eco-transformation of Industries Matrix, allows for the categorisation of industries or sectors in terms of environmental activity and carbon emissions.

Organisations were identified and selected by the research team in collaboration with Cambridgeshire County Council based on their regional significance in terms of their employment and economic contribution. These organisations were cross referenced against the top 100 companies listed in Cambridgeshire Limited 2019 (Grant Thornton UK LLP, 2019) and mapped by standard industry code (SIC) to the sectors included in the Eco-transformation of Industries Matrix (Kapetaniou and McIvor, 2020) (Figure 3). Eleven stakeholders, representing nine organisations and seven industry sectors in the Cambridgeshire and Peterborough region were selected (see Table 1).

**Table 1.** Study participants (by organisation and sector, ordered by interview date).

Organisation	Industry/Sector (SIC)	Short name
Cambridge University Hospitals NHS Foundation Trust	Human health and social work activities	HSW
G’s	Agriculture, forestry and fishing	AFF
Baker Perkins Ltd	Manufacturing	Manu
Redgate Software	Information and communication	I&C
Turners (Soham) Ltd	Transportation and storage	T&S
Chiltern Cold Storage Group Ltd	Transportation and storage	T&S
Hill	Construction	Construction
Abcam	Professional, scientific, and technical activities	PST
Marshall Aerospace and Defence Group	Manufacturing	Manu

They represent many of the key employers in the study region. However, many sit within the ILO’s upper circle (EGSS) and would likely have been omitted from any study relating only to green jobs. This research therefore sheds a light on the significant employment transitions taking place in organisations who are not specifically focused on delivering ‘green’ products or services.

Most of the individuals interviewed, (i.e. the participants) were executive level management and identified as key stakeholders in the recruitment, training, management and retention of people within their organisations. This research study received ethics approval in December 2020 before any interviews commenced. Participants gave informed consent, and their involvement was voluntary with no remuneration.

As well as representing the four categories of the Eco-Transformation of Industries Matrix, the interviewees also represented a range of organisations in terms of nearest postal town (Cambridge, Peterborough, Ely and Newmarket), size (turnover from £1million to >£100million), tenure (from 22 to 118 years trading), workforce size (11 to >501 whole time equivalents (WTEs) except for <10 whole time equivalents), geographical footprint (UK to global), impact of COVID-19 (Low to high) and number of vacancies (0 to >50). However, under-represented were small organisations (0–10 WTEs) and organisations established within the last 20 years.

Baseline data were gathered via an online pre-interview survey and then interviewees were followed up via email and telephone to secure appointments to undertake detailed interviews via online platforms (MS Teams and Zoom) using semi-structured techniques. Interviews took place between 16th December 2020 and 12th February 2021. Interviews lasted between 40 min and 120 min depending on the availability of the participants and content of discussions. A framework for the detailed, semi-structured interviews was used to support discussions, as well as a number of verbal and visual prompts – these were consistently used with all participants. The framework for the semi-structured interviews was as follows:

• Vacancies
• Employment/growth
• Region
• Organisation
• Impact of COVID-19
• Skills – needs, mapping, training
• Other

In an effort to uncover what is required by the workforce at different levels of the organisation, a series of matrices were developed to prompt participants to provide a one-word descriptor or phrase to describe the knowledge, skills, competencies and attributes their organisation felt were important. The prompts included the following ‘domains’: What do they need to know; what do they need to be able to do; what do they need to be like; and what/how do they need to think? Agreed definitions for knowledge, skills, competency and attributes were also provided as necessary to support the interview process.

All interviews were recorded and transcribed, anonymised and then analysed using thematic content analysis (Braun and Clarke, 2012). This coding was undertaken by the same researcher that carried out the semi-structured interviews. The coding process and nomenclature emerged organically from an increasing familiarisation with or ‘progressive focusing’ (Schutt, 2006) on the data set in recognition of the main themes of the discussions. This process was carried out using the NVivo 12 qualitative analysis software package. The codes were then aggregated into sub-themes and themes within the context of the framework of the semi-structured interviews (see Appendixes 2 and 3).

Coded content varied from 168 to 424 references per interview and the number of codes used ranged from 29 to 42. There was no direct correlation between the amount of coded content, the range of codes generated (so breadth of content covered) and the length of the interview, so broadly speaking there was equity between the perspectives represented by the participants.

## Results and discussion

At a global scale, all countries engaged in the Organisation for Economic Co-operation and Development (OECD) and European Centre for the Development of Vocational Training (CEDEFOP) interagency working group (IWG) on the social and employment dimensions of a greener economy recognise the importance of environmentally sustainable growth (Martinez-Fernandez et al., 2014; Maclean et al., 2018). The policy literature on research into the skills implications of the transition to a low carbon economy recognises the need for this to impact all job levels (ILO, 2011). This research shows that this is also recognised by employers at the local level, in the Cambridgeshire–Peterborough region of the UK. All participants in this study, regardless of their categorisation by the Eco-Transformation of Industries Matrix, recognised the importance of a transition to a greener economy and society or of ‘going green’. The strategic alignment of organisations with this agenda, however, varies depending on their sector, the external and internal drivers, culture of the organisation and its management.

### Positive approach to going green

None of the interviewees in this study identified what they would call green jobs within their organisation, yet all felt their organisation were making a contribution to a low carbon future to some extent.

‘*It is a big focus for our industry in general’* (T&S)

**Figure 4.**
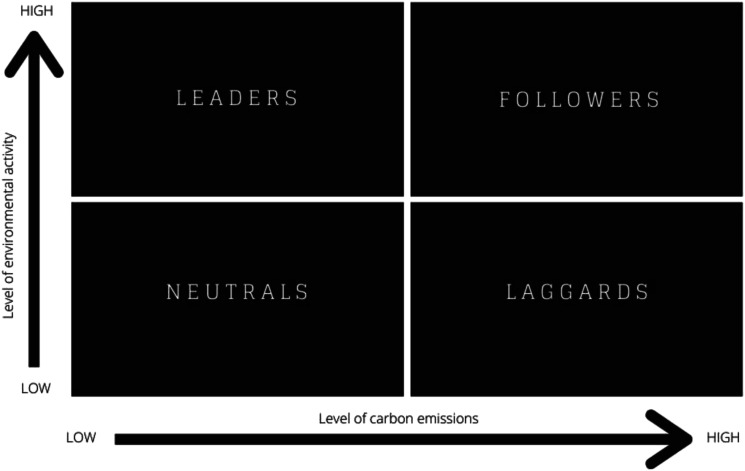
Eco-Transformation of Industries Matrix (adapted from Kapetaniou and McIvor, 2020).

In the pre-interview survey participants were asked to plot their own organisation’s position on the Eco-Transformation of Industries Matrix (Figure 4). This question was supported by a graphic image of the matrix and a description of the axes. See Appendix 1 for more details about the pre-interview survey questions.

This two-by-two matrix with the variables marked accordingly – the level of carbon emissions and environmental activities – was provided as a visual prompt. Outlined in Table 2 below is a comparison between the categorisation based on their sector in the *Going Green* report (Kapetaniou and McIvor, 2020) and the participants’ own responses.

**Table 2.** Comparative categorisation of organisations’ according to the Eco-Transformation of Industries Matrix (Kapetaniou and McIvor, 2020).

Organisation	Going Green categorisation by sector	Pre-interview survey categorisation by organisation
Cambridge University Hospitals NHS Foundation Trust	Neutrals	Leaders
G’s	Followers	Leaders
Baker Perkins Ltd	Followers	Neutrals
Redgate Software	Neutrals	Neutrals
Turners (Soham) Ltd	Laggards	Followers
Chiltern Cold Storage Group Ltd	Laggards	Neutrals
Hill	Followers	Leaders
Abcam	Leaders	Neutrals
Marshall	Followers	Followers

Over half of the interviewees estimated their organisation’s position on the matrix was further towards the top left than Kapetaniou and McIvor’s (2020) categorisation based on their industry sector. The trend was particularly apparent in traditional brown sector organisations that are taking a proactive approach to environmental initiatives and could be regarded as leaders in their sector, or where their sector categorisation perhaps fails to recognise the nuances of the organisations’ purpose.

Interviewees were also asked to consider whether they saw going green as a threat or opportunity to their organisation. Many recognised it as an opportunity and often also as a driver for organisational development and business improvement.

“*Opportunity, massive opportunities here*…” (HSW)

However, the majority felt that it offered both and most referred to opportunities and challenges (rather than threats). ‘*I think there is an opportunity, but I think it also brings up some challenges in terms of our supply chain*’ (Manu).

Interviewees were asked to comment on factors which are currently influencing their organisations’ focus on going green. These factors have been analysed in terms of where they originate from – internal or external to the organisation. A summary of these drivers is outlined in Table 3. The thematic analysis shows that internal drivers, for example, employees, customers, business improvement and organisational culture are more important than external drivers, for example, government policy/targets and/or industry regulations in some organisations.

**Table 3.** Summary of key drivers for change or going green.

	Key driver	Explanation
**External**	Policy	The push from government to move their sector towards going green
	Industry bodies	The push from industry bodies to move their sector towards going green
**Internal**	Stakeholders	Alignment with their organisational values
	Employees	Alignment with their personal values
	Customers	Align their personal and/or organisational values
	Business improvement	Opportunity to realise potential improvements in efficiency and operational excellence

### Vacancy rates static and sector specific

Vacancy rates for the majority of organisations represented in the study were static and predictable, driven by factors which are sector specific. For all sectors, growth was given as a reason for any increasing vacancy rates. The impact of COVID-19 was specifically noted in several sectors (HSW, Manu. and AFF). Interestingly the impact of COVID-19 was reported to have decreased vacancy rates with respondents in the Manufacturing and Human Health and Social Work sectors who noted the significance of remote working.

“50% of our admin team are now working from home, if not more” (HSW)

At a national scale, government policy can influence vacancy rates positively or negatively. Whilst the impact of Brexit was not a specific focus of this study, its impact on recent immigration policy was mentioned as leading to increases in vacancies (in AFF, HSW and T&S). The importance and positive effect of additional support for training, development and apprenticeships was mentioned (HSS, Manu. and Construction) and there was also some speculation on the potential impact of any changes to UK university fees in the future.

Most of the respondents related the vacancy rate to hard to fill roles, which were specific to each sector. These hard to fill roles included *‘…technology roles, finance roles, marketing roles, HR roles….’* (PST), *‘Nurses that are trained in over 150 bed hospitals’* (HSW), *‘…roles like product marketing roles, product management roles’ (*I&C), *‘…drivers…logistics – traffic and planning’* (T&S), *‘Skilled engineers’* and *‘…finance and senior roles’* (Manu.), and *‘Engineers – big kit and operational excellence’* (AFF).

As discussed, the negative impact of vacancies and skills needs on organisations is difficult to measure and often hidden, but as well as the financial cost of temporary staff, participants also recognised the impact on their existing workforce

### Reported skills needs

In terms of anticipating future skills needs, some responses were broadly positive in terms of believing skilled staff would become more available (HSW) whilst others felt skills needs would remain about the same as currently (I&C and T&S). The majority, however, shared specific concerns in certain domains and at different levels (AFF, Construction, PST, Manu. and T&S). These anticipated future skills deficits occurred in domains currently viewed as strategically important to the organisations interviewed.

There was also recognition that there is a difference between a skills shortage and an experience shortage. Respondents emphasised the importance of work experience, sector experience and/or experience of working in an equivalent setting.

All interviewees recognised the role of education, training and development in the upskilling and retraining of the workforce, with requirements specific to sector, current skills needs, and anticipated skills needs. Kapetaniou and McIvor (2020) suggest that the participation rate of employees in adult learning varies between about 21% and 11% depending on industry sector. The engagement rates reported by interviewees in this study varied by sector, with most suggesting their engagement rates were towards the high end of this estimate with only transport and storage tracking the lower limit of this estimate. Participants were asked what they considered was driving their workforce’s engagement with adult education in their organisation. Responses varied but it was again, like going green, driven by both internal and external factors, and motivated by the culture of the organisation, the type of workforce (e.g. technical/specialist) or pressure from stakeholders (e.g. employees).

### Identifying green skills

In an analysis of labour force characteristics that emerge as a response to the growing importance of environmental sustainability Consoli et al. (2016) identify the differences between green jobs and non-green jobs. Green skills can be defined as ‘*technical skills, knowledge, value, and attitude needed by green workers to perform tasks that contribute to sustainable environment, economy, and social development’* (Strietska-Ilina, et al., 2012 p. 103). Although the term ‘green skill’ is sometimes used interchangeably with other terms, such as low carbon skills (Ismail, et al., 2020), Aitchison (2015) notes that they share the common goal of contributing to the environmental protection and natural resources conservation. A green skill can be broken down into three parts; knowledge, ability and attitude/value and must also promote sustainable social, economy and environment (Ismail et al., 2020; Zubir et al., 2021). Essex and Hirst (2011) and Sern et al. (2018) both focus on the knowledge and ability aspects of green skills, and specifically for green industry (the overlap between circles 1 and 2 in Figure 1) and identify and prioritise these (Table 4 and Table 5).

**Table 4.** The elements of green skills (adapted from Sern et al., 2018).

No.	Element
1	Design skills
2	Energy skills
3	Communication skills
4	Procurement skills
5	Leadership skills
6	City planning skills
7	Waste management skills
8	Financial skills
9	Management skills
10	Landscaping skills

**Table 5.** Ranking of green skills required by industries (adapted from Essex and Hirst, 2011).

Ranking	Element
1	Design skills
2	Energy skills
3	Client skills
4	Leadership and management skills
5	Community skills
6	Construction skills
7	Town and country planning skills
8	Waste skills
9	Procurement skills
10	Landscape and environmental skills
11	Building management skills
12	Financial skills
13	Transport infrastructure skills

Despite representing all four domains of the Eco-Transformation Matrix, none of the participants in this study specifically identified shortages in these green skills which suggests they may only be relevant to organisations involved in the production of environmental goods or services, or that they failed to recognise the importance of the attitude/values aspect of green skills.

In order to identify the key skills currently required by organisations represented in this study, as well as their relative importance and future requirements, each interviewee populated a series of matrices with the help of the interviewer. In general, participants found this exercise challenging. Focusing on the differences between knowledge, abilities, attributes and experience drew out some interesting findings in terms of the requirements of the workforce by sector and organisation, now and as anticipated for the future. These are summarised in Table 6 at different levels of the organisation.

**Table 6.** Summary of key characteristics by level.

Level	Key characteristics
Entry	Primarily focus is personal and behavioural attributes
Management	Requirement is for individuals who can inspire, lead and understand the dynamics of team working. Recognition that new skill sets will be required in the future
Senior	Self-awareness, presentable, credible and authentic
All levels	Cultural fit is as important as previous knowledge, skills and experience

For entry level staff, our data suggest that organisations are primarily looking for personal and behavioural attributes. More important than their inherent knowledge was how individuals apply this to the workplace, for example, in the context of organisation procedures, process and business/IT systems. The ability to think logically or practically, being open, communicative, collaborative and willing to learn was often mentioned. At management level, our interviewees suggested that their organisations are looking for individuals who can inspire, lead and understand the dynamics of team working. There was recognition that new skill sets will be required by the managers of the future, understanding the needs of diverse teams and an acknowledgement that there are intergenerational differences in individuals’ motivations and perspectives which can potentially impact on the teams’ effectiveness. At an executive level, interviewees reported particular challenges in sourcing the right skills and again even more than at other levels ‘finding the right person’ with cultural fit was considered as important as previous knowledge, skills and experience. Specific skills noted included being able to think critically and laterally, forward plan, manage risk, lead for the future and network inside and outside the organisation.

These results are perhaps not surprising given that Zolkifli, et al., (2016) note that, even in the EGSS sector (at least in Malaysia) employers are likely to choose employees based not only on their knowledge and technical skills (hard skills) but that the ability to manage oneself and others (employability or generic skills) is also an important consideration.

Participants were aware that there were specific skills and experience required within their sector. For example, in manufacturing themes included apprentices, lack of training provision and issues with the levy for companies without their own in-house apprenticeship training. Whereas other organisations recognised that they may be classed as a sector with a low-skilled workforce and there might be limited opportunities for internal development within their organisation. Many participants also raised the role of culture and employee commitment within an organisation or sector, especially, for example, in manufacturing, agriculture, forestry and fishing.

In the literature, it is suggested that a balance of generic skills (e.g. autonomy and communication), generic green skills (such as reducing waste and improving energy and resource efficiency) and ‘topping up’ existing job-related skills is much more important to developing a low-carbon economy than more specialised, green skills (CEDEFOP, 2018). This is backed up by the results of this study, where specific skills were considered by many interviewees as less important than attitude and work ethos and therefore sit within the right-hand side of Figure 2 (above), and in particular the bottom right quadrant. This is important for employers looking to future proof their ‘workforces', and there was an indication that employers are taking greater responsibility for the skills needs of their employees. It is also important for the education and training sector, which should be mindful of the time-lag involved in responding to employers' needs.

In terms of anticipating future needs for skills, some responses were broadly positive in terms of skills being more available (HSW) others about the same as currently (I&C and T&S), but the majority shared specific concerns in certain domains and at various levels (AFF, Construction, PST, Manu. and T&S). These anticipated future skills deficits are in domains currently viewed as strategically important to the organisations interviewed and in organisations considered strategically important regionally.

### Role for technical and vocational education and training (TVET)

Education plays a major role in green economy skills, social skills and environmental skills development but in the context of lifelong learning, other stakeholders include employers, individuals and policymakers. (WEF, 2020) suggest that on average, employers expect to offer reskilling and upskilling to just over 70% of their employees by 2025. However, they note that employee engagement in those courses is lagging, with only 42% of employees taking up employer-supported reskilling and upskilling opportunities. In the report, *Green Skills, and Innovation for Inclusive Growth*, it is recommended that: *‘Countries need to balance and empower TVET systems to act as catalysers of greening jobs and skills and all jobs need to be part of the process, not just those linked to renewable energies but all sectors and at all levels....’* (OECD/CEDEFOP, 2014 p.16).

**Figure 5.**
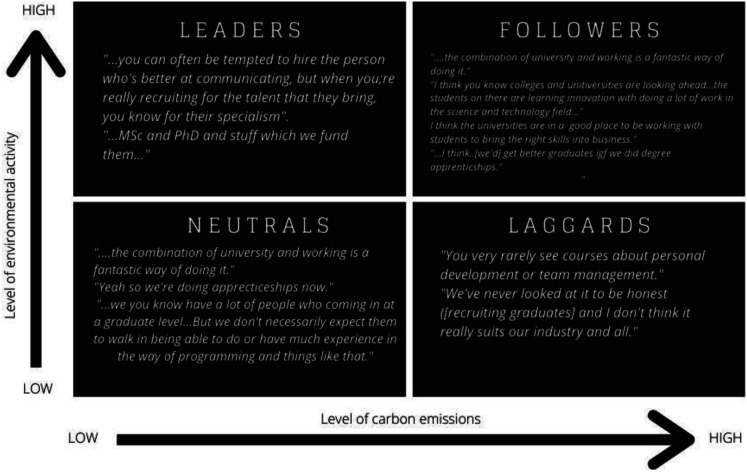
Participants quotes regarding higher education mapped to their sector’s categorisation on Eco-Transformation of Industries Matrix (adapted from Kapetaniou and McIvor, 2020).

The role of graduate training and apprenticeships was discussed with interviewees in this study and the extent to which it could offer a solution to the current and anticipated skills need was again found to be dependent on sector and the domain of skills need. The responses, mapped out in Figure 5 based on Kapetaniou and McIvor’s (2020) Eco-Transformation of Industries Matrix, show a range of perspectives, dependent on whether the organisation offers graduate opportunities and/or works in partnership with the sector on vocational training and apprenticeships. Some interviewees felt that courses failed to keep pace with industry whilst others saw universities and colleges as innovators.

In most sectors, it was acknowledged that work experience and social learning would be more important for the workforce of the future than specific knowledges, except in the case of Human health and social care (clinical) and Manufacturing (engineering).

For example, ‘…the level of clinical skills might be less, but the technical skills might be higher’ (HSW). This general finding is broadly in line with TVET literature and supports the use of the competency-based model for green skills which has provided a framework for this study (Pavlova, 2016).

### Upskilling and retraining is everyone’s responsibility

In their description of lifelong learning Maclean et al. (2018) include a wide variety of ways in which individuals who are seeking work or who are in employment continue to learn and to acquire the skills and competencies which impact their employment and earnings. Adams (2012) points out that measuring and tracking the skills acquired is difficult, given the range of competencies sought by employers, the diversity of ways in which skills and competencies are acquired, and the variations in duration, level and quality of the training being given. Certainly, reliable and comparable statistics on within-industry training are difficult to find (Maclean et al., 2018).

In this study, there was a general recognition by interviewees that access to good training providers is vital to upskilling and retraining, however, they suggest it should be a multi-stakeholder responsibility in the context of current and future challenges. It was broadly acknowledged that there was a general trend towards training becoming the responsibility of employers. Some participants raised this as an opportunity for inter-organisational networks to address some of the challenges of upskilling and retraining the workforce to support a low carbon post-COVID-19 transition to a cleaner, fairer economy and society. For example, *‘I think there’s certainly some things we can learn from them because they’ve got a very successful apprenticeship programme’* (Manu).

### COVID-19 – a disruptor

Many participants reflected on the last year as a period of consolidation with the anticipation of, and plans for, growth in 2022 and beyond. Many of the organisations interviewed have been significantly impacted by COVID-19, with over half of participants (55.6%) rating the impact as high. The impact of COVID-19, however, was not necessarily viewed as negative by participants in this study. Although they reported that it had impacted growth, in many cases organisations managed to remain profitable. Although COVID-19 was not explicitly recognised as driving their organisation’s transition to going green, interviewees did acknowledge COVID-19 as a disruptor. This disruption was discussed with participants in the context of its impact – positive or negative and level of impact – high, medium or low. A summary of their responses is outlined in Table 7.

**Table 7.** Positive and negative aspects of COVID-19 on organisations according to level of impact.

Low impact		Medium impact		High impact	
Positive	Negative	Positive	Negative	Positive	Negative
*‘It didn’t impact our essential operations so we carried on trading’. ‘But then we saw growth in our biopharma customers instead and we and we’ve been able to get some of our products to be used’. ‘We maintained our employees and we kept hiring. It was probably our biggest ever hiring year. We took advantage of it to hire more people’*		*‘This is a positive because we didn't make anybody redundant. Yes, we furloughed a number of staff but not masses and we got back to work as quickly as we could’.* *‘When we got the first lockdown the volume of traffic dropped by 19%. Miles per gallon increased quite dramatically. The level of risk generated by drivers reduced by over 65 percent....So from that point of view it actually made our operation more efficient, and I’ll imagine many other for just the companies the same’.*	*‘From a negative point of view, we have lost some very good people’.* *‘*[*we work in] very specific areas that can be harder hit by a recession’.*	*‘And then on the sort of positive side, it’s that there’s like new approaches. Senior management are listening to staff as well’.* *‘…in fact it is allowed us to restructure our business and we are stronger’.* *‘You get a different view from different people in the business. I think it’s been broadly positive…it should make us less geographically concerned’.* *‘…we’ve probably got a more highly engaged workforce on site than we had pre-Covid. But that isn’t replicated for the people working off site, really, but there have been other gains for people working off site.’* *‘...we are lucky and our business particularly is global….so we are much more insulated against things like this because we aren’t particularly biased towards any sector’.*	*‘And well, we couldn’t recruit our seasonal people from abroad because borders were closing what happened yeah’.* *‘…we had a much higher turnover than we would generally have. It meant that we had to recruit 500 more people than we would normally this summer yeah…those we recruited were only 60% as productive as predicted’.* *‘Can’t see an ending, so the morale is quite low’.* *‘We did furlough 90% of the staff/workforce…we did make 31 staff redundant in August’.* *‘Completely changed how we were working before’*

Interviewees were broadly confident in the ability of their organisation’s current workforces’ competences and capabilities to respond to the current requirements of the organisation, and the evidence they gave for this was very broad. They did, however, identify with many of the disruptors in Table 7, in the context of workforce trends, skills needs and trends in skills. For example, some referred to the geographical divide in the cost of living in the Cambridgeshire region limiting recruitment in some sectors (PST and HSW). One commented that:

“We can never relocate a person [to Cambridge]. It's too expensive!” (PST).

But another noted that an increase in agile and remote working is opening up a global labour market. For example, ‘*We’re working now competing at a global stage rather than … [a] Cambridge stage’* (I&C). Interviewees also recognised the role of the individual in upskilling and retraining or life-long learning as well as the employer and education providers. One said: ‘*I think you know self-directed learning is such a big key too…’* (AFF) Another said*: ‘…our people love focusing on their own development’*. (PST)

Some recognised the challenges of managing generationally diverse teams acknowledging the additional skills required by current managers saying ‘…*it is getting more and more important for managers and being able to manage teams that are far more diverse in terms of age*’ (Manu). These comments should also be of interest to the education sector, as the 2019 Auger report acknowledged, *‘The future challenges of technological innovation, artificial intelligence and shorter job cycles will require greater labour market flexibility. The post-18 education system needs to respond to this: doing more of the same will not be enough*’ (Auger, 2019: p. 18).

## Conclusion

The shift to a low-carbon economy implies structural changes across all sectors and occupations. As new occupations arise or grow in demand new green jobs will be created. It is these new green jobs which are the focus of much of UK transition policy. However, the broadest change is likely to involve existing jobs which are realigned to focus on what the ILO calls ‘environmentally friendly processes’ (Figure 1) even within the so-called brown sector. It is these that form the focus of this study.

Both new and greener jobs create new skills needs or require existing skills to be applied in new directions. As Keep (2020 p.6) points out *‘...Wind turbine service engineers are essentially electrical engineers with some additional skills around safe working at height’.* For almost all existing roles and professions (including HR, procurement, finance, and marketing as well as operations and manufacture), softer skills such as having an understanding of the need and knowledge of ways to reduce resources use and waste and taking steps to moving towards a circular economy will create ‘green’ skills needs in almost all occupations, levels of hierarchy and sectors (CEDEFOP, 2018).

Focusing on green jobs, and in particular new green jobs, therefore grossly underestimates the skills needs of a future workforce able to deliver a transition to a more sustainable low-carbon economy. In this study, of a selection of employers significant to the regional economy of Cambridgeshire and Peterborough, none fitted the profile of ‘producing green products or services’ yet all have a role to play. This study has highlighted some of the complexities of addressing the green skills from an employer perspective.

Within the context of the impact of COVID-19, policymakers are committed to a green recovery, as well as the public and business support for ‘going green’ through initiatives like ‘Build Back Better’. However, a green recovery requires more than policies to drive low carbon economic activity. In particular, it needs investment in people and skills (Aldersgate, 2020). It is clear from this study that education is critical, but that providers also need to engage with organisations more closely to anticipate and offer the support employers require in the upskilling and retraining of their workforces. Recognising the organisational turn from knowledge-based skills towards values- and attitudes-based skills the technical and vocational education and training (TVET) sector needs to reflect this through their learning and teaching provision.

COVID-19 is recognised as both a disrupter and a catalyst in this study. The impact has obviously been significant for many sectors involved but its potential to precipitate changes could be seen as an opportunity to expedite the social change and reskilling and upskilling of the workforce required to support any transition to a cleaner, fairer economy and society. However, there is still a lack of research providing evidence of the effectiveness of a green stimulus or about the impact of COVID-19 on the transition. Therefore, policymakers need to be cognisant that the disruption to organisations caused, in this case by COVID-19, cannot be necessarily separated from the prevailing economic and societal dynamics affecting the workforce such as the great divide, skills activism, innovation adaption, the shrinking middle and the four-generational workplace. This may equally apply to the disruption caused by Brexit.

As outlined by the participants in this research study, an inclusive low carbon, post-COVID-19 transition to a cleaner, fairer economy and society will require contributions from all sectors of the economy, not just the Environmental Goods and Services Sector (EGSS) and any unwarranted focus on green jobs is likely to result in industry leaders becoming disenfranchised. Regardless of their sector’s position on the Eco-transformation of Industries Matrix, individual business leaders, in this one UK region at least, recognise the importance of a green recovery, albeit predicated by economic growth. This finding may be of particular application to areas where there are significant numbers of employers in sectors outside the EGSS but are strategically important to post-Covid recovery and transition targets. In this study, we have highlighted the need to focus beyond the scope of green jobs to fully realise the potential contribution all sectors can make to help tackle the disruption of COVID-19 and to turn this into an opportunity to progress towards a sustainable low carbon future.

## Figures and Tables

**Figure A1.**
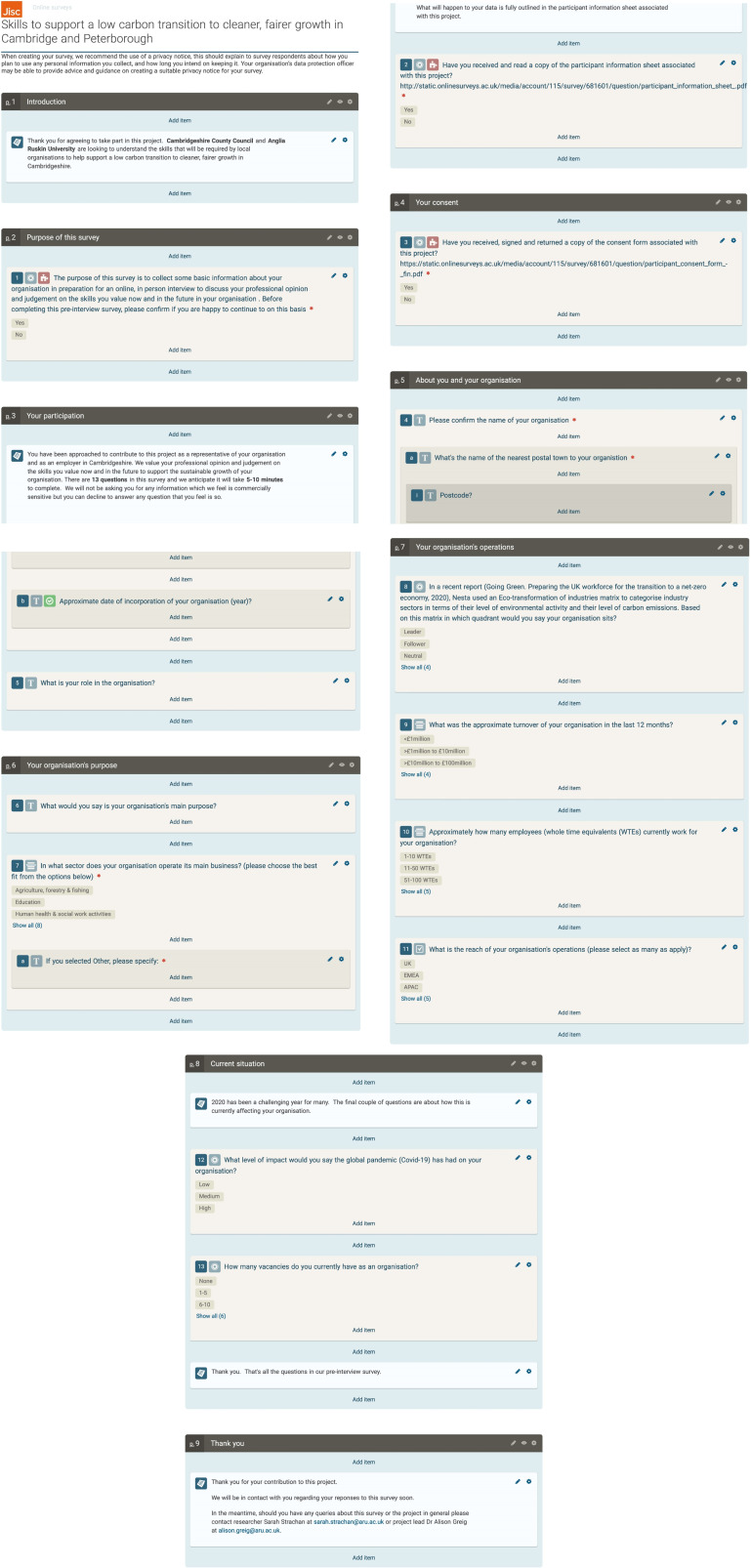
Pre-interview survey.

## Appendix 2 NVivo 12 – pre-aggregated coding structure.

**Table:**

Adult education drivers
Adult education rate
Apprenticeships – levy
Automation and new technologies
Build back better
Business improvement
Commuting
Declining vacancy rate
Difficult to fill vacancies
Digital transformation
Drivers for change
Drivers for green
Drivers for vacancy rate
Easy to fill vacancies
EGSS
Flexible or remote working
Furlough
Geographical footprint
Government agenda
Graduates
Green neutral
Green opportunity
Green threat
Impact of covid
Impact of skills need
Impact of vacancies
Increasing vacancy rate
Inter-company collaboration
Knowledge – attributes
Local government
Local infrastructure
Local networks
No vacancies
Pay awards
Redundancies
Re-skilling and up-skilling
Sector experience
Sector role levels
Skills – attributes
Skills – experience
Skills shortage
Skills – labour market
Static vacancy rate
Talent acquisition
Training and development
Trends in skills need
Trends in workforce and growth
Workforce confidence

## Appendix 3 NVivo 12 – aggregated coding structure.

**Table:**

Drivers
Automation and new technologies
Business improvement
Digital transformation
Drivers for change
Government agenda
Education, training and development
Adult education drivers
Adult education rate
Apprenticeships
Graduates
Training and development
Geography
Commuting
Global to local
Local government
Local infrastructure
Local networks
Inter-company collaboration
Green agenda
Build back better
Drivers for green
EGSS
Green neutral
Green opportunity
Green threat
Impacts
Impact of covid
Furlough
Pay awards
Redundancies
Impact of skills need
Impact of vacancies
Sector
Sector experience
Sector role levels
Skills
Knowledge – attributes
Re-skilling and up-skilling
Skills – attributes
Skills – experience
Trends in skills
Skills shortage
Skills – labour market
Trends in vacancies
Drivers for vacancy rate
Vacancies
Difficult to fill vacancies
Easy to fill vacancies
Vacancy rates
Declining vacancy rate
Increasing vacancy rate
No vacancies
Static vacancy rate
Trends in workforce
Flexible or remote working
Talent acquisition
Workforce and growth
Workforce confidence
